# G-Quadruplex DNAzyme Molecular Beacon for Amplified Colorimetric Biosensing of *Pseudostellaria heterophylla*

**DOI:** 10.3390/s130101064

**Published:** 2013-01-16

**Authors:** Zhenzhu Zheng, Jing Han, Wensheng Pang, Juan Hu

**Affiliations:** 1 Institute of Drug Research, Fujian Academy of Chinese Medicine, Fuzhou 350003, China; E-Mails: smilepearl727@gmail.com (Z.Z.); drjinghan@gmail.com (J.H.); 2 The College of Pharmacy, Fujian University of Traditional Chinese Medicine, Fuzhou 350122, China; E-Mail: pws@fjtcm.edu.cn (W.P.); 3 The Second People's Hospital of Fujian Province, Fuzhou 350003, China

**Keywords:** G-quadruplex DNAzyme, molecular beacon, colorimetric, *Pseudostellaria heterophylla*

## Abstract

With an internal transcribed spacer of 18 S, 5.8 S and 26 S nuclear ribosomal DNA (nrDNA ITS) as DNA marker, we report a colorimetric approach for authentication of *Pseudostellaria heterophylla* (PH) and its counterfeit species based on the differentiation of the nrDNA ITS sequence. The assay possesses an unlabelled G-quadruplex DNAzyme molecular beacon (MB) probe, employing complementary sequence as biorecognition element and 1:1:1:1 split G-quadruplex halves as reporter. In the absence of target DNA (T-DNA), the probe can shape intermolecular G-quadruplex structures capable of binding hemin to form G-quadruplex-hemin DNAzyme and catalyze the oxidation of ABTS^2−^ to blue-green ABTS^•−^ by H_2_O_2_. In the presence of T-DNA, T-DNA can hybridize with the complementary sequence to form a duplex structure, hindering the formation of the G-quadruplex structure and resulting in the loss of the catalytic activity. Consequently, a UV-Vis absorption signal decrease is observed in the ABTS^2−^-H_2_O_2_ system. The “turn-off” assay allows the detection of T-DNA from 1.0 × 10^−9^ to 3.0 × 10^−7^ mol·L^−1^ (R^2^ = 0.9906), with a low detection limit of 3.1 × 10^−10^ mol·L^−1^. The present study provides a sensitive and selective method and may serve as a foundation of utilizing the DNAzyme MB sensor for identifying traditional Chinese medicines.

## 1. Introduction

*Pseudostellaria heterophylla* (PH), a common traditional Chinese medicine, has been utilized to treat night sweats, hyperirritability, asthenia after illnesses, various lung and spleen diseases [1–4]. However, many counterfeit species, e.g., *Lophatherum gracile* (LG), *Liriope platyphylla* (LP), *Ophiopogon japonicus* (OJ), *Stemona sessilifolia* (SS) and *Stemona japonica* (SJ) [5], are misused in the market owing to their lower cost or erroneous identification. The counterfeit species may have weaker or completely different pharmacological actions compared with the authentic counterpart, and thus the authentication is the key to ensure the therapeutic effect and raise consumers' confidence in Traditional Chinese Medicine [6]. Nowadays, analysis of well-characterized marker compounds is the most popular method for identifying the herbal species utilizing HPLC [7,8], HPCE [9], GC-MS [10,11], NIR spectroscopy [4], *etc.* However, in many herbal medicines the chemical compounds change with external environment and processing conditions, which diminishes the reliability of these identification methods. Genetic composition is unique for each individual herbal species and is less influenced by environmental and processing factors. As reported, internal transcribed spacer of 18S, 5.8S and 26S nuclear ribosomal DNA (nrDNA ITS) has proven to be a particularly valuable resource for phylogenetic analysis in many angiosperm families [12–16]. Nowadays, many genetic tools have been utilized for the authentication of herbal species, e.g., RAPD [17], RFLP [18], MASA [18], MAS-PCR [19]. The practicality of these methods varies with their sensitivity, reliability and cost of execution [20]. Hence, the development of a sensitive, reliable and low-cost analytical method based on DNA sequences is desirable. G-quadruplex molecular beacon (MB) DNAzyme, a label-free and amplified detecting assay, fulfills all these requirements.

Since their discovery in 1996 [21], molecular beacons (MBs) have been extensively utilized in biological applications [22,23]. MBs are initially designed with a hairpin shape, featuring a stem-loop structure [21,24]. While the stem part is usually built with Watson-Crick hydrogen bonding between natural DNA base pairs, the 5′- and 3′-ends of a MB structure are labeled with a fluorophore and a quencher, respectively [25,26]. However, the employment of the fluorophore and quencher results not only in high costs, but also complexity of processes [26].

G-quadruplex DNAzyme, formed by the folding of four G-rich repeats and binding with hemin, possesses a property mimicking horseradish peroxidase (HRP) [27–30] and catalyzes the H_2_O_2_-mediated oxidation of the colorless 2,2′-azino-bis(3-ethylbenzthiazoline-6-sulfonic acid) diammonium salt (ABTS^2−)^ into the blue-green radical anion ABTS^•−^[31–34] to achieve signal amplification in DNA detection [35]. Moreover, the unusual G-rich nucleic acid has been found to possibly split the whole sequence [33,36] and lead to an extraordinary selectivity for the detection of nucleic acids [37], proteins and other biomolecules [31,38]. Also, the utilization of split G-quadruplex makes the assay design more flexible [39].

Wang and co-workers have employed a split G-quadruplex DNAzyme tethered to both the ends as reporter instead of the fluorophore/quencher combination, which avoids any complex labeling process [26]. Considering these advantages, we have also combined the split G-quadruplex structure and MB technique to develop a label-free G-quadruplex DNAzyme MB oligonucleotide sensor for biosensing of PH nrDNA ITS sequences. In the sensor, a novel 1:1:1:1 split mode of G-quadruplex is utilized and one GGG repeat is tethered to both the ends as the reporter. The complementary sequence for target PH nrDNA ITS sequence locates at the loop portion and serves as sensing element.

## 2. Experimental

### 2.1. Materials

All the oligonucleotides were first synthesized and purified by Sangon Biotechnology Co., Ltd. (Shanghai, China). The working solutions (10^−4^ mol·L^−1^) of oligonucleotides were prepared in 25 mM Tris-HAc buffer (pH 7.0) and stored at 4 °C until used. Hemin was purchased from Aladdin Reagents (Shanghai, China) and the stock solution was prepared in dimethyl sulfoxide (DMSO). H_2_O_2_ was purchased from Fuchen Chemical Reagents (Tianjin, China) and ABTS was purchased from Aladdin Reagents. Before used, H_2_O_2_, ABTS, and hemin working solutions were prepared in working buffer (pH 7.5) containing 50 mM Tris-HCl, 150 mM NH_4_Cl, 20 mM KCl, 0.03% Triton X-100 and 1% DMSO. All reagents were used as received without further purification.

### 2.2. Instrumentation

A Perkin Elmer (Lambda 750) UV-vis-NIR spectrophotometer was used to collect the UV-vis absorbance spectra of the ABTS^2−^-H_2_O_2_ reaction system. Circular dichroism (CD) experiments were performed on an Aviv Model 420 spectrometer (Aviv, Lakewood, NJ, USA).

### 2.3. Assay Procedure

In a typical assay, 0.5 μL of probe (5.0 × 10^−5^ mol·L^−1^) was hybridized with different concentrations of T-DNA in Tris-HOAc buffer (25 mM) for 40 min at room temperature. After that, 0.8 μL of hemin solution (6.25 × 10^−5^ mol·L^−1^) was added and the mixture was incubated for 60 min. Then, 20 μL of ABTS (2.5 × 10^−2^ mol·L^−1^), 2.5 μL of H_2_O_2_ (0.2 mol·L^−1^), and the working buffer (50 mM Tris-HCl, pH 7.5, 150 mM NH_4_Cl, 20 mM KCl, 0.03% Triton X-100 and 1% DMSO) were added to adjust the volume to 250 μL. After 5 min, the UV-vis spectrum was collected on a Perkin Elmer UV-vis-NIR spectrophotometer at room temperature and the photo was taken immediately. The maximum absorption wavelength of the oxidation product ABTS^•−^ was about 419 nm.

### 2.4. CD Measurements

In the circular dichroism (CD) experiments, 2.5 μL of probe-1, probe-2, probe-3, probe-4 (1.0 × 10^−4^ mol·L^−1^) in the absence of T-DNA were respectively added to 247.5 μL of working buffer (50 mM Tris-HCl, pH 7.5, 150 mM NH_4_Cl, 20 mM KCl, 0.03% Triton X-100 and 1% DMSO) and incubated for 60 min. In the T-DNA-containing system, 2.5 μL of probe-4 (1.0 × 10^−4^ mol·L^−1^) and 2.5 μL of T-DNA (1.0 × 10^−4^ mol·L^−1^) were first mixed together and incubated for 40 min. After that, 245 μL of the working buffer was added to the mixture and then also incubated for 60 min. Finally, the CD spectra were measured on an Aviv Model 420 spectrometer at room temperature. The wavelengths were scanned from 220 to 320 nm, with a step of 2 nm and bandwidth of 1 nm.

## 3. Results and Discussion

### 3.1. Principle of the DNAzyme MB Sensor Detecting PH nrDNA ITS Sequence

According to the differentiation between PH and its counterfeit species in nrDNA ITS sequence, a section of the nrDNA ITS sequence specific for PH works as target and is labeled as T-DNA. The nrDNA ITS sequences specific for the counterfeit species are labeled as C-DNA.

**Figure 1.**
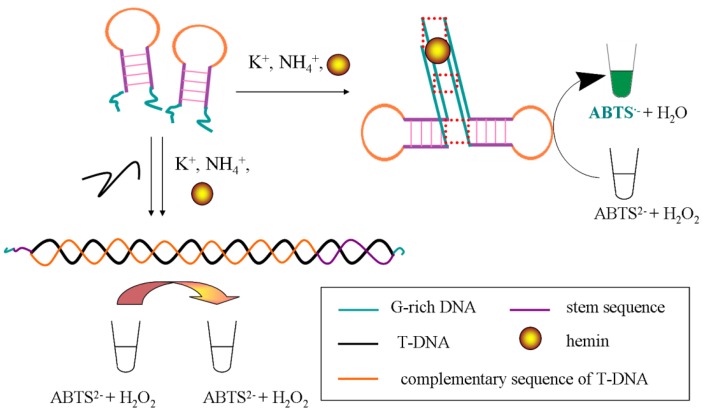
Working principle of the 1:1:1:1 split G-quadruplex DNAzyme MB sensor for PH nrDNA ITS.

As shown in Figure 1, the complementary sequence for T-DNA locates at the hairpin loop and serves as the sensing element. In the absence of T-DNA, two oligonucleotide probes shape an intermolecular G-quadruplex structure through the association of the GGG repeats. Then hemin is able to specifically bind to the G-quadruplex with high affinity and yield a DNAzyme which can catalyze the oxidation of ABTS^2−^ by H_2_O_2_ to the colored product ABTS^•−^ revealed by a strong UV-vis absorption signal. In the presence of T-DNA, the loop of the MB sensor is combined and the stem opens to form a duplex DNA structure hindering the formation of the intermolecular G-quadruplex DNAzyme and leading to the loss of the catalytic activity. As a result, a weaker UV-vis absorption signal can be observed in the ABTS^2−^-H_2_O_2_ reaction system.

### 3.2. Design of Probes

In this work, four types of probe were designed. Probe-1 was designed with the middle sequence (orange characters) complementary to T-DNA and both ends possessing two GGG repeats (green characters) according to the frequently used 2:2 split mode. Furthermore, an insertion of seven nucleotides (violet characters) for hairpin stem structure was applied in probe-2. Probe-3 was designed with the middle sequence (orange characters) complementary to T-DNA and one GGG repeat (green characters) locating at both ends according to the fresh 1:1:1:1 split mode. Then probe-4 was designed by further inserting seven nucleotides (violet characters). All the oligonucleotides were listed in Table 1.

### 3.3. Optimization of Probe

**Figure 2.**
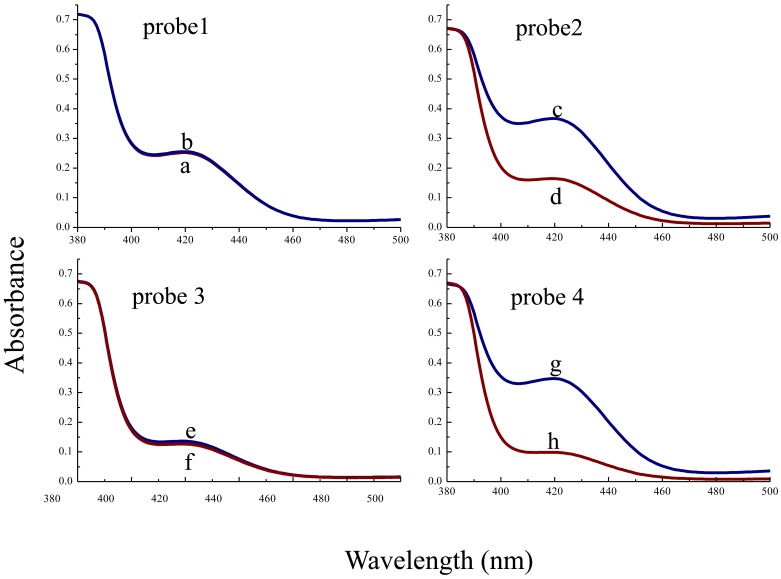
Absorbance spectra of the H_2_O_2_ mediated ABTS^2−^ colorimetric system. (**a**) probe-1 (1.0 × 10^−7^ mol·L^−1^); (**b**) probe-1 (1.0 × 10^−7^ mol·L^−1^), T-DNA (1.0 × 10^−7^ mol·L^−1^); (**c**) probe-2 (1.0 × 10^−7^ mol·L^−1^); (**d**) probe-2 (1.0 × 10^−7^ mol·L^−1^), T-DNA (1.0 × 10^−7^ mol·L^−1^); (**e**) probe-3 (1.0 × 10^−7^ mol·L^−1^); (**f**) probe-3 (1.0 × 10^−7^ mol·L^−1^), T-DNA (1.0 × 10^−7^ mol·L^−1^); (**g**) probe-4 (1.0 × 10^−7^ mol·L^−1^); (**h**) probe-4 (1.0 × 10^−7^ mol·L^−1^), T-DNA (1.0 × 10^−7^ mol·L^−1^).

As introduced in Section 3.2, probe-1 and probe-2 were designed with 2:2 G-quadruplex split mode while probe-3 and probe-4 were designed with 1:1:1:1 split mode. Probe-2 and probe-4 had a loop-stem structure by inserting seven nucleotides while probe-1 and probe-3 did not. To obtain an optimum oligonucleotide probe, the catalytic activity of the four probes was investigated and compared under the same conditions. Figure 2 shows that the UV-vis absorbance spectrum of the probe-1 system has nearly no change induced by T-DNA while a decreasing rate of 223% induced by T-DNA is observed in the probe-2 system.

Meanwhile, the absorbance signal of the probe-3 system decreases a little, and a rate of decrease of 351% induced by T-DNA is observed in the probe-4 system. From the results, we can find that the probe-3 system and probe-4 system have larger decrease rates than the probe-1 and probe-2 systems, respectively. This is because while binding with T-DNA both probe-1 and probe-2 with 2:2 split mode could still assemble to form the G-quadruplex structure more easily than probe-3 and probe-4, respectively, and the middle G-quadruplex DNAzyme is less disrupted. Therefore, the probes with 2:2 split mode have stronger background signals.

Meanwhile, the probe-2 system and probe-4 system both possessing hairpin structure have larger decrease rates than the probe-1 system and probe-3 system, respectively. That may be because the stem part of a hairpin structure promotes the probes into shaping G-quadruplex structures and makes the G-quadruplex structure more difficult to dissociate in the absence of T-DNA. As a result, probe-4 with 1:1:1:1 split mode and hairpin structure is chosen as the optimum probe in the whole experiment.

### 3.4. CD Spectra of Probes

It has been reported that CD spectrum of a typical parallel G-quadruplex structure has a positive peak at 265 nm and a negative band near 240 nm, and that of a typical antiparallel G-quadruplex structure shows a positive peak near 295 nm and a negative peak close to 265 nm [40], while that of a B form duplex DNA structure has a positive peak at 277 nm and a negative peak at 245 nm [41]. To demonstrate the secondary structure shapes in the four probes, CD spectra of the probes were measured in the presence or absence of T-DNA.

**Figure 3.**
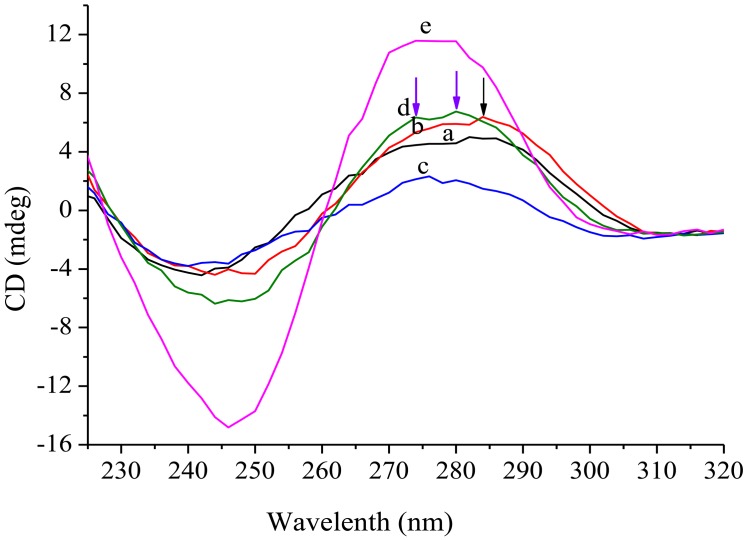
CD spectra of (**a**) probe-1 (1.0 × 10^−6^ mol·L^−1^), (**b**) probe-2 (1.0 × 10^−6^ mol·L^−1^), (**c**) probe-3 (1.0 × 10^−6^ mol·L^−1^), (**d**) probe-4 (1.0 × 10^−6^ mol·L^−1^), (**e**) probe-4 (1.0 × 10^−6^ mol·L^−1^) + T-DNA (1.0 × 10^−6^ mol·L^−1^).

From the spectra in Figure 3, we can find that all the probe systems have a negative band around 245 nm, and there is some shift in the positive band. Probe-1 (line a) exhibits a positive peak at around 282 nm, probe-2 (line b) at 284 nm and a shoulder peak at around 274 nm, probe-3 (line c) at around 276 nm, and probe-4 (line d) has a positive peak at 280 nm and a shoulder peak at around 274 nm. The results indicate that all four probes have the ability of shaping a G-quadruplex structure. The weakest CD signal of probe-3 system suggests the least G-quadrupex shape. Furthermore, the engineered stem part induces probe-2 and probe-4 to show a shoulder peak at 274 nm. In the presence of T-DNA, the CD spectrum of probe-4 exhibits a larger positive peak around 277 nm, suggesting the existence of a B form duplex DNA structure. This may be due to the principle that the added T-DNA combines the loop section of probe-4 to form a duplex structure.

### 3.5. Colorimetric Analysis of T-DNA

**Figure 4.**
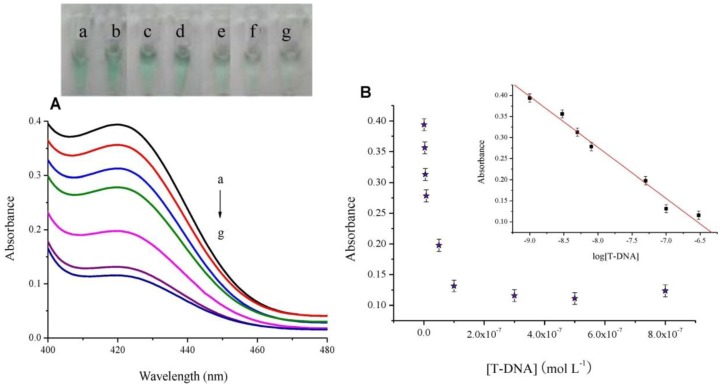
(**A**) Absorbance spectra of the H_2_O_2_ mediated ABTS^2−^ colorimetric system with probe-4 (1.0 × 10^−7^ mol·L^−1^); T-DNA (a) 1.0 × 10^−9^; (b) 3.0 × 10^−9^; (c) 5.0 × 10^−9^; (d) 8.0 × 10^−9^; (e) 5.0 × 10^−8^; (f) 1.0 × 10^−7^ and (g) 3.0 × 10^−7^ mol·L^−1^. Inset: visual color changes of the systems with different concentrations of T-DNA. (**B**) Absorbance value at 419 nm of the colorimetric system with probe-4 (1.0 × 10^−7^ mol·L^−1^) *versus* T-DNA concentration. Inset: derived calibration curve.

According to the general procedure mentioned in Section 2.3, we have utilized the biofunctional probe-4 to analyze different concentrations of T-DNA in the range of 1.0 × 10^−9^ to 8.0 × 10^−7^ mol·L^−1^ by collecting the absorbance spectra and colorimetric signals of the colored product ABTS^•−^. As shown in Figure 4(A), in the presence of 1.0 × 10^−9^ mol·L^−1^ T-DNA, the ABTS^2−^-H_2_O_2_ reaction system promoted by the G-quadruplex DNAzyme MB probe shows a strong absorbance and colorimetric signal (curve a, picture a). As the increase of T-DNA concentration, UV-vis absorbance (curve b to g) and colorimetric signal (picture b to g) both decrease gradually. The above results indicate that in the presence of T-DNA, T-DNA hybridizes with the loop part to form a duplex structure and blocks the formation of the middle intermolecular G-quadruplex DNAzyme, resulting in a loss of catalytic activity.

As shown in Figure 4(B), the maximal absorbance value at 419 nm decreases obviously with an increasing concentration of T-DNA and then levels off after 3.0 × 10^−7^ mol·L^−1^. Additionally, the inset is the calibration curve for quantitative analysis. It shows that the absorbance is linearly dependent on the logarithm of T-DNA concentration in the range from 1.0 × 10^−9^ to 3.0 × 10^−7^ mol·L^−1^ (R^2^ = 0.9906). Furthermore, the average absorbance values for the blank systems (n = 8) are tested to be 0.3804 (RSD = 4.79%). The detection limit (3σ/slope) is estimated to be 3.1 × 10^−10^ mol·L^−1^. The results indicate that the G-quadruplex DNAzyme MB sensor can be a sensitive oligonucleotide assay for identifying T-DNA.

### 3.6. Specificity Study

To investigate the selectivity of the G-quadruplex DNAzyme MB sensor for PH nrDNA ITS, control experiments were done as follows: nrDNA ITS sequences of the main counterfeit species (C-DNA) in 1.0 × 10^−7^ mol·L^−1^ were respectively added to the sensor systems, and their absorption signals were collected.

**Figure 5.**
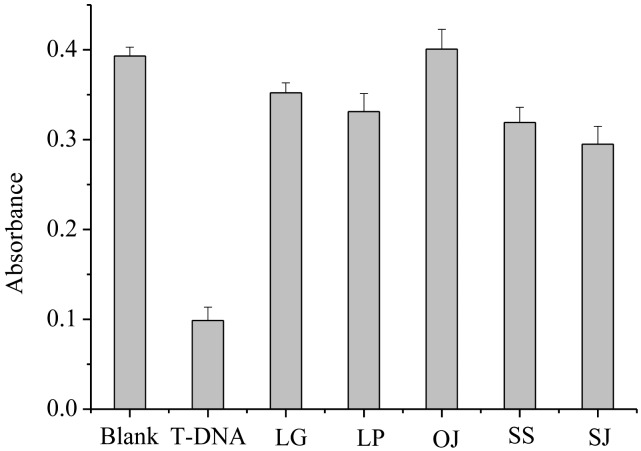
Absorbance value at 419 nm of the G-quadruplex DNAzyme MB colorimetric system containing probe-4 (1.0 × 10^−7^ mol·L^−1^) in the presence of T-DNA (1.0 × 10^−7^ mol·L^−1^) or C-DNA (1.0 × 10^−7^ mol·L^1^) respectively.

As shown in Figure 5, only the presence of T-DNA makes the absorbance signal decrease largely and there is little decrease in the presence of C-DNA. The above results imply that C-DNA nearly cannot hinder the formation of the intermolecular G-quadruplex DNAzyme, and thus has no interference in the detection of T-DNA. We can conclude that the label-free G-quadruplex DNAzyme MB sensor can serve as a novel selective oligonucleotide assay for authentication of PH and its adulterants.

## 4. Conclusions

In the present study, we have combined a novel split mode 1:1:1:1 of G-quadruplex with the MB technique to build a label-free turn-off G-quadruplex DNAzyme MB sensor for authentication of PH and its counterfeit species with nrDNA ITS sequence as DNA marker. In the sensor, two probe-4 units shape an intermolecular G-quadruplex DNAzyme in the middle possessing high catalytic activity. The presence of T-DNA hinders the formation of the G-quadruplex DNAzyme and thus the decrease of catalytic activity revealed by weaker colorimetric and absorbance signals. Through the strategy, the oligonucleotide sensor shows high sensitivity and selectivity toward target T-DNA. To the best of our knowledge, this work provides the first example of development of a G-quadruplex DNAzyme MB sensor for discrimination of PH and its counterfeit species. We believe this work may serve as a foundation of further design and utilization of the DNAzyme MB sensor for authentication of Traditional Chinese Medicines.

## Figures and Tables

**Table 1.** Sequences of the oligonucleotides used in the assembly of the G-quadruplex DNAzyme MB sensor.

**Oligomer**		**Sequence (from 5′to 3′)**
T-DNA	PH	TCGAAACCTG CCCAGC- - - - AGAACGACCA GCGAACA
C-DNA	LG	TCG TGACCCT T - - AAC - - - - AA AACAGACC GCGCACG
	LP	TCAACACGTG TGCAGTTTAG AGCATACT CA ATA AACA
	OJ	TCAATACGTG TG - AGTTTA - AGCATACTCA ATA AACA
	SS	TCAATACATG TGCAGTTTA - AG CATACTCA GTGAACA
	SJ	TCAGTACATG TGCAGTTTAG AGCATA -TCA ATA AACA
probe	probe-1	GGGATT GGGATT TGTTCGCTGGTCGTTCTGCTGGGCAGGTTTCGA TTAGGG TTAGGG
	probe-2	GGGATT GGGATT TGTTCGC TGGTCGTTCTGCTGGGCAGGTTTCGA GCGAACA TTAGGG TTAGGG
	probe-3	GGGATT TGTTCGCTGGTCGTTCTGCTGGGCAGGTTTCGA TTAGGG
	probe-4	GGGATT TGTTCGC TGGTCGTTCTGCTGGGCAGGTTTCGA GCGAACA TTAGGG
	Hum24	(TTAGGG)_4_
